# Spatiotemporal Dynamics of Emerging Foot-and-Mouth Disease, Bluetongue, and Peste Des Petits Ruminants in Algeria

**DOI:** 10.3390/v17071008

**Published:** 2025-07-17

**Authors:** Ilhem Zouyed, Sabrina Boussena, Nacira Ramdani, Houssem Eddine Damerdji, Julio A. Benavides, Hacène Medkour

**Affiliations:** 1 Institute of Veterinary Sciences, University Constantine 1 Frères Mentouri, Constantine 25100, Algeria; ilhem.zouyed@umc.edu.dz (I.Z.); houss.dm@gmail.com (H.E.D.); 2 Management of Animal Health and Productions Laboratory, University Constantine 1 Frères Mentouri, Constantine 25100, Algeria; s_boussena@umc.edu.dz; 3 Regional Veterinary Laboratory of El Oued, National Institute of Veterinary Medicine, El Oued 16000, Algeria; drnaciraramdani@gmail.com; 4 MIVEGEC, University of Montpellier, IRD, CNRS, 34394 Montpellier, France; julio.benavides@ird.fr; 5 Doctorado en Medicina de la Conservación y One Health Institute, Facultad de Ciencias de la Vida, Universidad Andrés Bello, Santiago 8320000, Chile

**Keywords:** Africa, FMD, BT, PPR, emerging viral diseases, livestock, national surveillance, data analysis, bioclimatic hotspot

## Abstract

Foot-and-mouth disease (FMD), bluetongue (BT), and Peste des Petits Ruminants (PPR) are major emerging and re-emerging viral infections affecting ruminants. These diseases can threaten livestock health, food security, and economic stability in low- and middle-income countries, including Algeria. However, their dynamics remain mostly unknown, limiting the implementation of effective preventive and control measures. We analyzed outbreak data reported by Algerian veterinary authorities and the WAHIS database from 2014 to 2022 for FMD; from 2006 to 2020 for BT; and from 2011 to 2022 for PPR to investigate their spatiotemporal patterns and environmental drivers. Over these periods, Algeria reported 1142 FMD outbreaks (10,409 cases; 0.16/1000 incidence), 167 BT outbreaks (602 cases; 0.018/1000), and 222 PPR outbreaks (3597 cases; 0.096/1000). Small ruminants were the most affected across all diseases, although cattle bore the highest burden of FMD. BT primarily impacted sheep, and PPR showed a higher incidence in goats. Disease peaks occurred in 2014 for FMD, 2008 for BT, and 2019 for PPR. Spatial analyses revealed distinct ecological hotspots: sub-humid and semi-arid zones for FMD and BT, and semi-arid/Saharan regions for PPR. These patterns may be influenced by species susceptibility, animal movement, trade, and climatic factors such as temperature and rainfall. The absence of consistent temporal trends and the persistence of outbreaks suggest multiple drivers, including insufficient vaccination coverage, under-reporting, viral evolution, and environmental persistence. Our findings underscore the importance of targeted species- and region-specific control strategies, including improved surveillance, cross-border coordination, and climate-informed risk mapping. Strengthening One Health frameworks will be essential to mitigate the re-emergence and spread of these diseases.

## 1. Introduction

Transboundary animal diseases (TADs) pose a serious threat to public health, food security, and the global economy. These diseases have far-reaching economic and social impacts on both animal and human populations, particularly in low- and lower-middle-income countries [1]. Africa is especially vulnerable, as many of its communities rely heavily on livestock for food, income, and livelihoods. In the northern part of the continent, especially the Maghreb region, livestock production is a cornerstone of rural economies [2], contributing between 40 and 80% of the Gross Agricultural Product [3]. However, this sector faces numerous challenges, including low farm productivity, technical limitations, and animal health issues [4]. TADs significantly hinder livestock development by limiting productivity, economic growth, and international trade [5]. Due to the transboundary nature of these diseases and the movement of animals across regions and continents, they pose a risk not only to individual countries but to entire regions as well [6].

Algeria, the largest country in the Maghreb, maintains a livestock population of over 38 million ruminants. Despite its status as an oil-producing nation, the Algerian economy is significantly supported by agriculture (including livestock), which accounts for 15% of total output [7]. The Algerian government has expressed a strong commitment to enhancing agricultural production and supporting rural development [8]. However, the country’s vast and diverse geography, inadequate rural sanitation, and extensive borders increase its vulnerability to TADs. Several viral TADs, listed by the World Organization for Animal Health (WOAH), are of particular concern in Algeria: Foot-and-Mouth Disease (FMD), Bluetongue (BT), and Peste des Petits Ruminants (PPR). BT and PPR are more recent concerns, first detected in 2000 and 2011, respectively [9,10], while FMD has been reported since 1966 [11]. Additional threats include emerging diseases such as sheep pox and lumpy skin disease. 

FMD is a severe and highly contagious disease that affects cloven-hooved animals [11]. It is caused by the Foot-and-Mouth Disease virus (FMDV), a member of the *Aphthovirus* genus, *Picornaviridae* family. FMDV circulates globally in seven geographically distinct reservoirs [12], with annual losses ranging from $5.5 to $21 billion due to production losses and vaccination costs [13]. PPR, caused by the Small Ruminant Morbillivirus (PPRV) of the *Paramyxoviridae* family [14], can cause mortality rates as high as 90% in sheep and goats. It has expanded across Africa, Asia, and parts of Europe, threatening over one billion small ruminants worldwide and causing annual losses of $1.45–2.1 billion [15,16]. BT is an arboviral disease that affects both domestic and wild animals, caused by the *Bluetongue virus* (BTV) of the *Orbivirus* genus (*Reoviridae* family), primarily transmitted by *Culicoides* midges, with estimated global annual losses of $3 billion [17].

Despite the significance of these diseases, data from Algeria remain scarce. Existing studies on FMD [11,18,19,20], PPR [10,21,22,23], and BT [24,25,26] are limited in scope and mostly predate 2017. These works have reported the circulation of FMDV serotypes O and A, BTV serotypes 1, 2, and 4, and PPRV lineage IV [26]. Control measures in Algeria have largely relied on vaccination, selective culling with compensation (for FMD), targeted vaccination in epizootic regions (for PPR), and vector control (for BT). However, comprehensive epidemiological data are lacking, and the factors driving the emergence and spread of these diseases remain poorly understood.

Presently, the absence of systematic spatiotemporal analyses constrains effective TAD control in Algeria. Understanding the geographic distributions and seasonal patterns of these outbreaks is essential for identifying high-risk periods and hotspots. This knowledge is critical for estimating economic impacts, improving disease preparedness, and designing targeted surveillance and intervention strategies to prevent and mitigate future outbreaks.

## 2. Materials and Methods

### 2.1. Study Area Description

**Figure 1:**
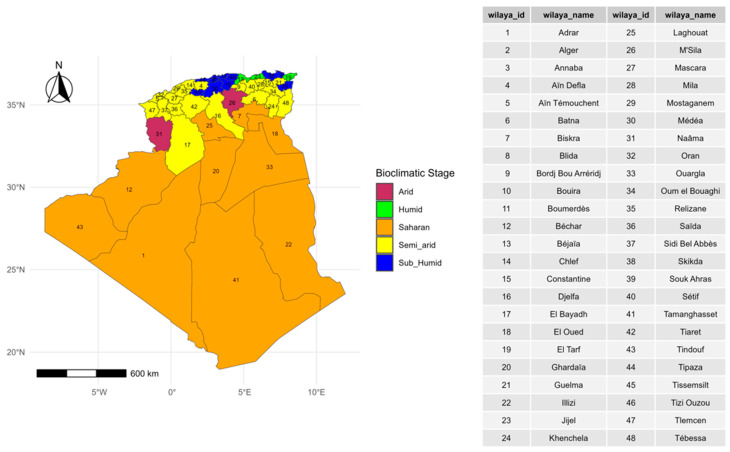
Distribution of bioclimatic stages by wilaya in Algeria.

Algeria, the largest country in Africa, is located in the north of the continent, extending from 18°58′ to 37°6′ N and 8°40′ W to 12° E. Its altitudes range from 40 m below sea level (Chott Melghigh) in El Oued to 2918 m above sea level (Tahat) in Tamanrasset. Currently, the country is divided into 58 administrative districts (wilayas), subdivided into 1541 municipalities (communes). However, at the time of the initial outbreaks, it was divided into only 48 districts (Figure 1). Most of the population and economic activity are concentrated in the northern coastal region, which covers less than 4% of the national territory. Livestock farming is dominated by sheep (81.2%, ~31 million), followed by goats (13%, ~5 million) and cattle (4.5%, 1.7 million; 52% of which are dairy cows) [7].

In Algeria, five Mediterranean bioclimatic domains are defined by Emberger’s pluviothermic quotient, which combines precipitation and temperature [27]. These domains are the humid, the sub-humid, the semi-arid, the arid, and the Saharan domains (Figure 1). Each wilaya is typically defined by a single climatic type. However, certain wilayas, such as Naama and M’sila, encompass two distinct types. In our analysis, we have focused on the dominant type, which covers the largest percentage of the area.

### 2.2. Data

#### 2.2.1. The Study Period

The data on the different diseases cover the following periods: from 2014 to 2022 for FMD; from 2006 to 2020 for BT; and from 2011 to 2022 for PPR.

#### 2.2.2. Data Sources

In Algeria, the primary method of disease surveillance and reporting is passive surveillance conducted by veterinary authorities, including public and private veterinarians. This approach involves monitoring and reporting diseases such as FMD, BT, and PPR. Upon detection, veterinarians are required to submit a written report to the relevant Wilaya Veterinary Inspector, the municipal authorities, and the national veterinary authority [28]. The National Institute of Veterinary Medicine (INMV) and its nine regional laboratories are responsible for the laboratory diagnosis and confirmation of outbreaks.

The FMD data were obtained from the Veterinary Services Direction (DSV) of the Algerian Ministry of Agriculture and Rural Development and Fisheries. These records include the year of occurrence, species affected, the wilaya reporting, the bioclimatic stage, the annual number of outbreaks, suspected and confirmed cases, and infection rate. The FMD outbreaks that occurred within the same year in a wilaya were grouped, with disease periods categorized as one to three months. Serotypes and vaccination data were sourced from the official WAHIS database (Table S1). The diagnosis was made based on clinical signs and was subsequently confirmed using RT-PCR at the Central Veterinary Laboratory or ELISA at the INMV regional laboratories. Samples were also sent to FMD reference laboratories (IZSLER, Brescia, Italy, and Pirbright Institute, Pirbright, UK) for confirmation and genotyping. The BT and PPR data were obtained directly from the OIE-WAHIS digital platform based on notifications by Algerian veterinary services. These datasets include the disease name, the species affected, the location, the outbreak scale, the control measures, and any laboratory tests in progress or completed. The data also included monthly and yearly occurrences, bioclimatic stage, susceptible animals, confirmed cases, deaths, and slaughtered animals (Table S1). Some outbreaks were presented in the database without a clear distinction of the species affected; hence, they were considered together in the analysis. As in the case of the FMD diagnosis, clinical cases of BT and PPR were diagnosed and confirmed through ELISA and RT-PCR at INMV laboratories across the country.

#### 2.2.3. Data Analyses

In this study, the spatiotemporal dynamics of these diseases were examined using different methods:

##### Description of Spatiotemporal Patterns

We estimated the total number of outbreaks (defined as a cluster of disease cases in the same time, area, and presenting similar clinical signs) and confirmed cases (defined as suspected cases with laboratory confirmation) by temporal unit (month, year), spatial unit (country level, wilaya level), and host species (cattle, goats, and sheep). The incidence rate was calculated as the number of cases/the number of animals in the population (per 1000 animals).

The same analyses were conducted for each of the three diseases and all host species reported in the database. To better understand potential ecological drivers affecting the spread of these diseases, we also compared the number of cases and outbreaks according to the five bioclimatic domains present in Algeria. All statistical analyses were conducted in R [29]. Maps and spatial analyses were created using the raster, sf, geodata, and ggplot 2 packages in R.

##### Spatiotemporal Ecological Regression Model

We built Poisson and negative binomial regression models to test the association between the annual number of cases and variables, including year, wilaya ID, latitude, longitude, affected species, bioclimatic zone, and seasonal quarter. Before model selection, variable multicollinearity was checked using Cramér’s V test for categorical variables and Pearson correlation for numerical variables. Variables were considered highly correlated if the coefficient was over 0.4 [30] or if the Pearson’s r value was over 0.7 [31]. We also assessed variance inflation factors (VIFs) to quantify collinearity and ensure reliable coefficient estimation. We refined the model using a stepwise selection method based on Akaike Information Criterion (AIC) to ensure an optimal balance between complexity and explanatory power.

The Poisson model’s overdispersion was assessed using the Cameron & Trivedi dispersion test [32]. If significant, negative binomial models were used instead, as they account for non-Poisson variation by introducing a dispersion parameter [33]. Model selection was based on LRT and AIC values [34], and only the best models with the lowest AIC were retained and shown in the results. The negative binomial model used a Pearson chi-square test to assess overdispersion. We assessed the model fit using the Pearson Chi-Square Goodness-of-Fit test and the Deviance test [33]. The DHARMa residual diagnostic test [35] was also used to assess model fit by simulating residuals and detecting deviations from expected distributions, including overdispersion, zero inflation, and non-normality.

##### Global Spatial Autocorrelation Analysis

Global spatial autocorrelation cluster analysis was performed to detect potential geographical patterns of disease spread in Algeria. We performed a spatial clustering analysis using the global Moran’s I statistic [36] to assess the distributions of cases across Algeria as random, clustered, or dispersed. Given the lack of exact GPS points of each outbreak, the coordinates of the centroid of the municipality affected were used as the case’s location. Moran’s I values range from −1 to 1, with positive values indicating clustering, negative values indicating dispersion, and values close to zero indicating random distribution. The analysis was performed for each year separately to identify possible temporal variations in spatial clustering. Global Moran’s I was calculated for individual years in the dataset. The resulting Moran’s I values and *p*-values were examined to determine whether spatial clustering was statistically significant.

##### Local Spatial Autocorrelation Analysis (LISA)

To identify high-risk (hotspot) and low-risk (coldspot) areas, we applied LISA, specifically Local Moran’s I [37]. Spatial analyses were performed using R v4.5.0 with the spdep package. Data were georeferenced based on case locations using the municipality’s centroid GPS coordinates, and spatial weight matrices were constructed using a k-nearest neighbor approach to define spatial relationships between locations.

## 3. Results

### 3.1. Disease Burden by Livestock Species

**Figure 2:**
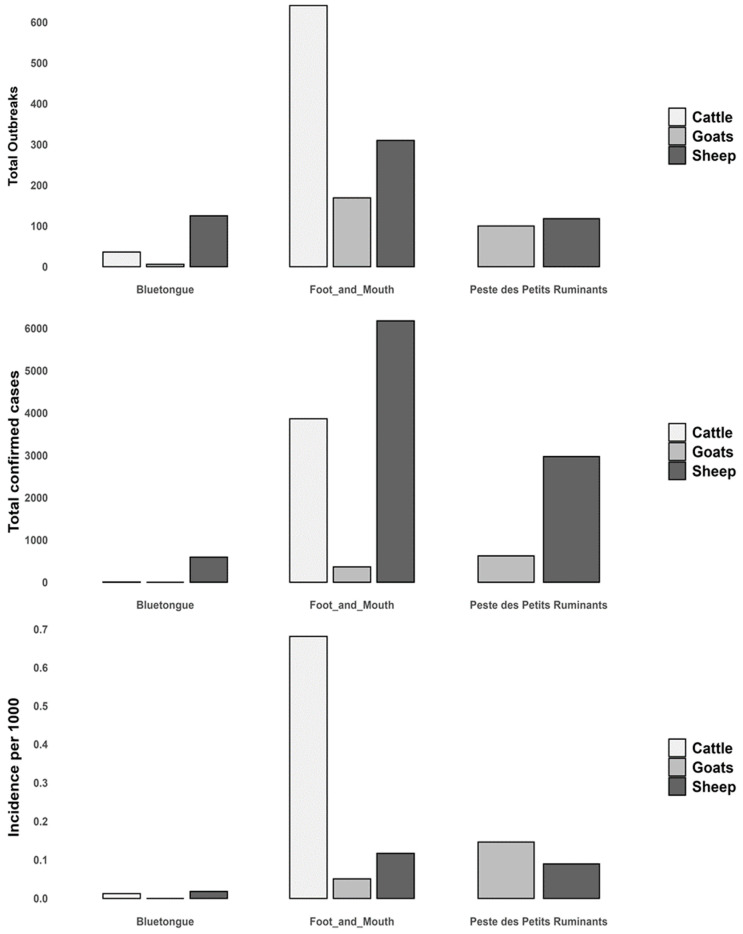
Number of confirmed cases, outbreaks, and incidence rates of FMD (2014–2022), BT (2006–2020), and PPR (2011–2022) in cattle, sheep, and goats in Algeria.

The total number of cases recorded was 10,403 (with an incidence rate of 0.16 per 1000 animals) for FMD, 609 (0.018) for BT, and 3597 (0.096) for PPR. Cattle were most affected by FMD (0.68), sheep by BT (0.018), and goats by PPR (0.15) (Figure 2).

### 3.2. Temporal Trend

**Figure 3:**
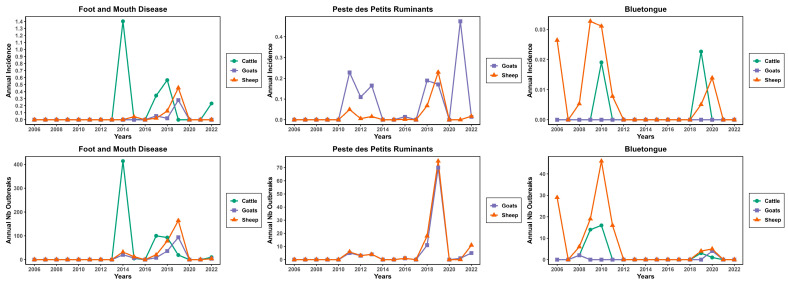
Annual trends number of FMD, BT, and PPR outbreaks and incidence rates in Algeria from 2006 to 2022, spanning three animal species. See also Table S2.

The temporal trends show distinct outbreak dynamics for each disease, with sharp peaks indicating years of major epizootics, particularly in sheep and cattle. In general, all three diseases showed different temporal patterns with major outbreak peaks in different years (e.g., 2008 for BT, 2014 for FMD, and 2018 for PPR) (Figure 3).

For FMD, the most prominent event was a massive outbreak in 2014, primarily affecting cattle with an annual incidence of 1.4. A second significant outbreak occurred between 2017 and 2019, also affecting sheep and goats, with incidence rates in 2019 of 0.45 and 0.3, respectively. After the peaks in 2014 and 2019, outbreaks decreased significantly and remained sporadic in subsequent years.

Concerning BT, after a first outbreak peak affecting sheep in 2006, a second substantial peak occurred between 2008 and 2011, primarily affecting sheep (incidence, 0.03), with low-level activity in cattle (incidence, 0.02), and lower in goats. A third, smaller resurgence of outbreaks occurred around 2019–2020, involving first sheep, with a lower level in goats, and cattle.

For PPR, minimal to no outbreaks were reported between 2011 and 2017. However, there was a significant increase in outbreaks around 2018–2019, with sheep being the most affected species (incidence, 0.25), followed closely by goats (incidence, 0.2). The disease continued spreading, and recent outbreaks reappeared in 2021–2022 in sheep and goats (incidence rate of 0.47 in goats). Except for these periods, outbreaks decreased significantly and remained sporadic in subsequent years (Figure 3).

### 3.3. Spatial Distribution

**Figure 4:**
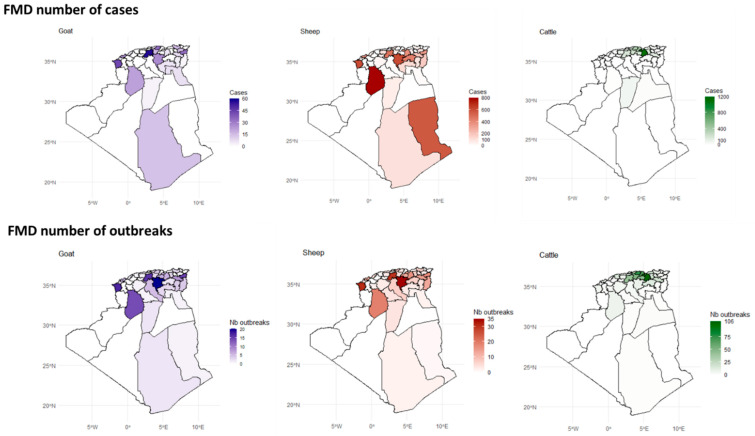
Spatial distribution of confirmed FMD cases and outbreaks in goats, sheep, and cattle across Algeria. Wilayas with no cases or outbreaks are shaded in white. Discrepancies in Wilayas with outbreaks reported and confirmed cases relate to outbreaks where laboratory confirmation was positive for the species in the area. See also Figures S1–S3.

The spatial analysis of outbreaks and confirmed cases data revealed that the distribution of FMD outbreaks varied greatly across the different districts, largely influenced by the animal species affected (Figure 4).

Between 2014 and 2022, a high number of FMD outbreaks in cattle were concentrated in the northern region of Algeria. This area, comprising six wilayas (Sétif, Bordj Bou Arréridj, Béjaia, Tizi Ouzou, Bouira, and Médéa), recorded between 50 and 106 outbreaks each, which is considered highly endemic. Among these, Sétif (northeastern) reported the highest number of outbreaks (n = 106) (Figure 4 and Figure S1).

FMD outbreaks in sheep were highest (30–35) in three northern wilayas: two in the north central region (M’sila and Médéa) and one in the far west coast (Tlemcen). El Bayedh, in the south-northern region (steppe), recorded the second-highest number of outbreaks (20–30), followed by four wilayas in the oriental steppe, Tébessa and Souk Ahras (in the Tunisian border); Oum El Bouaghi and Sétif; and Oran on the west coast.

The spatial distribution of FMD outbreaks in goats closely resembles that observed in sheep. However, the highest number of outbreaks was slightly lower, with the highest endemic risk being 15–20 outbreaks, concentrated in the north (Figure 4 and Figure S3).

**Figure 5:**
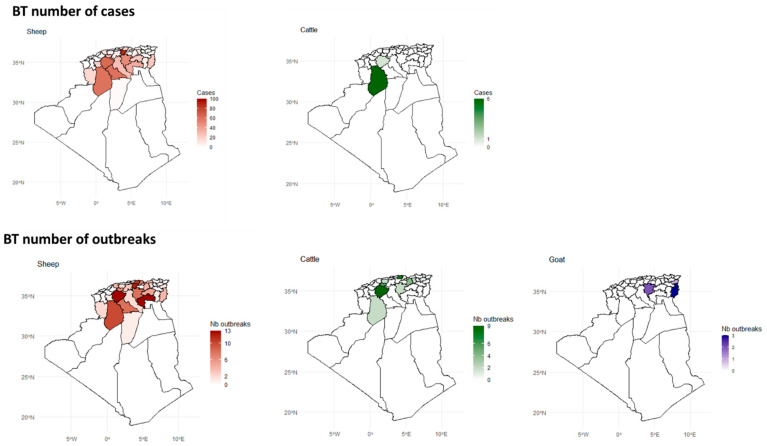
Spatial distribution of confirmed BT cases and outbreaks in goats, sheep, and cattle across Algeria. See also Figures S4–S6.

The distribution of BT in Algeria shows a clear species-specific and regional pattern. Sheep are the most affected species, particularly in the steppe (wilayas bordering the Tell Atlas to the north and the Saharan Atlas to the south), with El Bayadh being a hotspot for confirmed cases and outbreaks. Cattle are less affected, with cases and outbreaks largely restricted to El Bayadh. Goats show a different pattern, with cases concentrated at the country’s eastern border, such as Tébessa (Figure 5).

**Figure 6:**
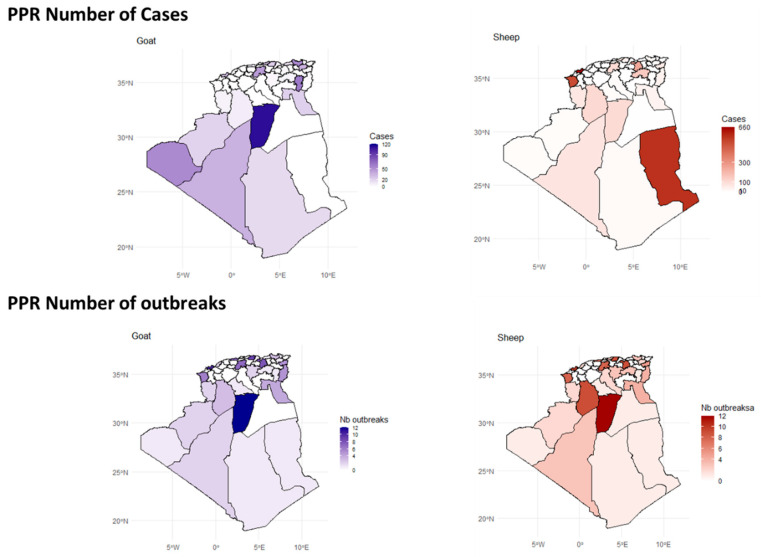
Spatial distribution of confirmed PPR cases and outbreaks in goats and sheep across Algeria. See also Figures S7 and S8.

PPR patterns indicate significant regional variations, with higher PPR activity observed in the north and north-Saharan regions among sheep, and a predominant impact in north-Saharan regions (Ghardaïa) among goats (Figure 6).

The highest number of confirmed PPR cases has been observed on the eastern coast (Oran and Tlemcen) and southeastern wilayas, particularly Illizi, which recorded up to 660 cases, making it the most affected region. These areas form a clear hotspot for PPR in sheep. The distribution of outbreaks relatively mirrors the case distribution. Ghardaïa (northern Sahara) was the most affected region (Figure 6 and Figure S7).

### 3.4. Bioclimatic Associations with Disease Outbreaks

**Figure 7:**
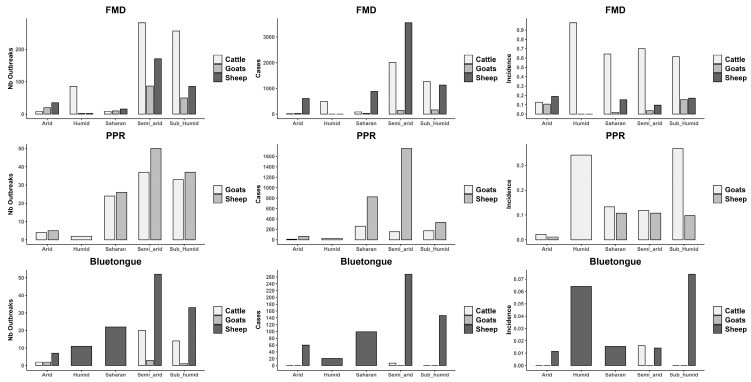
Bar charts illustrating the number of confirmed cases, number of outbreaks, and incidence rates of FMD, Bluetongue, and PPR in three livestock species: cattle, goats, and sheep. The data are categorized across various bioclimatic zones, highlighting the relationship between the distribution of the diseases and environmental conditions.

A comparison of the number of outbreaks, cases, and incidence rates of each disease per bioclimatic zone revealed that the sub-humid and semi-arid zones are hotspots for FMD and BT in all species (Figure 6), with sheep being the most affected, followed by goats and cattle. Sheep are the most affected species across all zones by FMD, particularly in the semi-arid and sub-humid zones, followed by goats, then cattle, where both cases and outbreaks peak significantly. With PPR, the highest number of cases and outbreak numbers in sheep and goats, respectively, are recorded in the semi-arid and Saharan zones (Figure 7).

### 3.5. Spatiotemporal Ecological Regression Model

The results indicating significant temporal and species-related variations in disease incidence are presented in Table 1.

#### 3.5.1. FMD

The disease occurrence was notably higher in 2014 (IRR = 29.08, 95% CI: 6.75–96.70, *p* < 0.0001) and 2018 (IRR = 17.47, 95% CI: 3.51–78.45, *p* < 0.001) compared to 2022, while other years did not show significant differences. Species also played a crucial role, as cattle (IRR = 12.57, 95% CI: 4.19–36.69, *p* < 0.0001) and sheep (IRR = 15.67, 95% CI: 7.76–31.34, *p* < 0.001) had significantly higher incidence rates compared to goats. Seasonal variations were less pronounced, with a slight but non-significant increase in the second half of the year (IRR = 16.83, 95% CI: 0.69–1492.24, *p* = 0.086) compared to the first half. Model diagnostics confirmed an adequate fit, as indicated by the non-significant Deviance test (*p* = 0.05) and Pearson’s Chi-Square test (*p* = 0.188), as well as an overdispersion statistic close to 1 (1.08). Additionally, AIC (1775.622) and DHARMa residual diagnostics further support the model’s reliability.

#### 3.5.2. BT

The results of the negative binomial Poisson model for BT cases suggest significant findings for some variables. The third [IRR: 0.03 (95% CI: 0.00–0.34, *p* = 0.006)] and fourth quarters [IRR of 0.02 (95% CI: 0.00–0.23, *p* = 0.0021)] revealed a significantly reduced incidence rate ratio (IRR) compared to the first quarter. However, sheep showed a highly significant association with BT cases (IRR = 155.53, 95% CI: 20.02–1208.25, *p* < 0.0001), indicating that sheep have a much higher risk of contracting BT compared to cattle.

The overdispersion statistic of 1.0118 and AIC of 306.91 suggest that the model fits the data adequately. Both the Pearson’s Chi-Square statistic (46.54, *p* = 0.4499) and the Deviance statistic (45.88, *p* = 0.4772) show no significant lack of fit, confirming the model’s adequacy. In addition, goodness of fit tests further tested the model’s reliability, with the DHARMa test showing no issues: the KS test *p*-value = 0.91409, dispersion test *p*-value = 0.696, outlier test *p*-value = 1, and Levene’s test for homogeneity of variance (n.s.) indicated a well-fitting model without heteroscedasticity, all indicating that there is no evidence of poor fit or significant outliers in the model. These results confirm that the model is well-suited to the data.

#### 3.5.3. PPR

The negative binomial model was selected for analyzing PPR cases due to its ability to handle moderate overdispersion, as indicated by the overdispersion statistic (1.52). Model fit assessment showed no significant lack of fit based on the deviance statistic (104.45, *p* = 0.056), supporting the adequacy of the model. While Pearson’s chi-square test suggested some lack of fit (125.99, *p* = 0.0016), this is likely due to overdispersion, which the negative binomial model appropriately addresses. The AIC of 761.27 further supports the model’s comparative performance. Diagnostic checks using DHARMa residual analysis confirmed the appropriateness of the model. The QQ plot residuals showed no significant deviation from normality (KS test: *p* = 0.44742), and no significant dispersion issues were detected (Dispersion test: *p* = 0.056). Additionally, outlier tests (*p* = 1) and Levene’s test for homogeneity of variance (n.s.) indicated a well-fitting model without heteroscedasticity.

The model also captures key epidemiological patterns (Table 1). It revealed significant seasonal variations in PPR incidence. Compared to quarter 3, PPR cases were significantly higher in quarter 1 (IRR = 3.81, 95% CI [1.21, 9.67], *p* = 0.010) and quarter 4 (IRR = 3.25, 95% CI [0.98, 9.04], *p* = 0.033), highlighting seasonal peaks. Regarding species susceptibility, sheep exhibited a significantly higher incidence of PPR cases compared to goats, with an IRR of 4.32 (95% CI [2.35, 7.88], *p* < 0.001).

Given these factors, the negative binomial model provides a robust and interpretable framework for understanding PPR case distribution, demonstrating an adequate fit while effectively addressing overdispersion. Therefore, this model was retained as the most appropriate choice for inference.

### 3.6. Global Spatial Autocorrelation Analysis

#### 3.6.1. FMD

The spatial autocorrelation analysis using Moran’s I statistic for the years 2014, 2015, 2017, 2018, and 2019 revealed no statistically significant clustering of incidence rates across the study area. For 2014, the Moran’s I statistic was slightly negative (−0.049) and not statistically significant (*p* = 0.55), indicating no evidence of spatial clustering. In 2015, the Moran’s I was strongly negative (−0.52), indicating a weak tendency toward spatial dispersion with a non-significant *p*-value of 0.72, again suggesting no spatial autocorrelation. The years 2017, 2018, and 2019 showed small negative Moran’s I values (−0.022 and −0.083 for 2017 and 2019, respectively) with non-significant *p*-values (0.29 and 0.64), while 2018 showed a positive Moran’s I (0.07) with a marginally lower *p*-value of 0.11, indicating a weak and non-significant trend toward spatial clustering. For 2022, the Moran’s I was negative (−0.117) with a *p*-value of 0.55, indicating no significant spatial autocorrelation.

#### 3.6.2. BT

The global Moran’s I test was applied to assess the spatial autocorrelation of BT incidence rates during outbreak years (2006, 2009, 2010, and 2011) to ensure the validity of results. In 2006, a positive Moran’s I value of 0.13 was observed, with a *p*-value of 0.071, suggesting a weak but not statistically significant spatial clustering. In contrast, 2009 exhibited a strong negative Moran’s I of −0.88, but this result was not significant (*p* = 0.933), indicating no meaningful spatial pattern. The year 2010 showed a very low positive Moran’s I (0.037) with a non-significant *p*-value (0.194), again suggesting no spatial autocorrelation. However, in 2011, a statistically significant positive Moran’s I of 0.42 was detected (*p* = 0.023), indicating a notable spatial clustering of incidence rates during that year. These results highlight that spatial clustering of outbreaks was most pronounced and statistically significant only in 2011.

#### 3.6.3. PPR

The incidence rate of PPR was calculated per 1000 head of affected species for each wilaya and year to evaluate the spatial distribution of the disease. Spatial autocorrelation was assessed using Moran’s I statistic, but only for years with a sufficient number of spatial units (wilayas) to ensure valid statistical inference. Based on this criterion, the years 2011, 2018, and 2019 were selected for analysis. In 2011 (Moran’s I = −0.0515, *p* = 0.292), 2018 (Moran’s I = −0.0706, *p* = 0.515), and 2019 (Moran’s I = −0.0012, *p* = 0.2553), the Moran’s I values were close to zero and statistically non-significant, indicating a random distribution of cases with no evidence of spatial autocorrelation. For 2022, a negative Moran’s I value was observed (−0.4723, *p* = 0.6988), suggesting a possible tendency toward spatial dispersion, although this result was also not statistically significant. Overall, these findings suggest that PPR incidence did not exhibit significant spatial clustering or dispersion across wilayas during the studied years, reflecting a spatially random distribution of outbreaks.

### 3.7. Local Spatial Autocorrelation Analysis (LISA)

**Figure 8:**
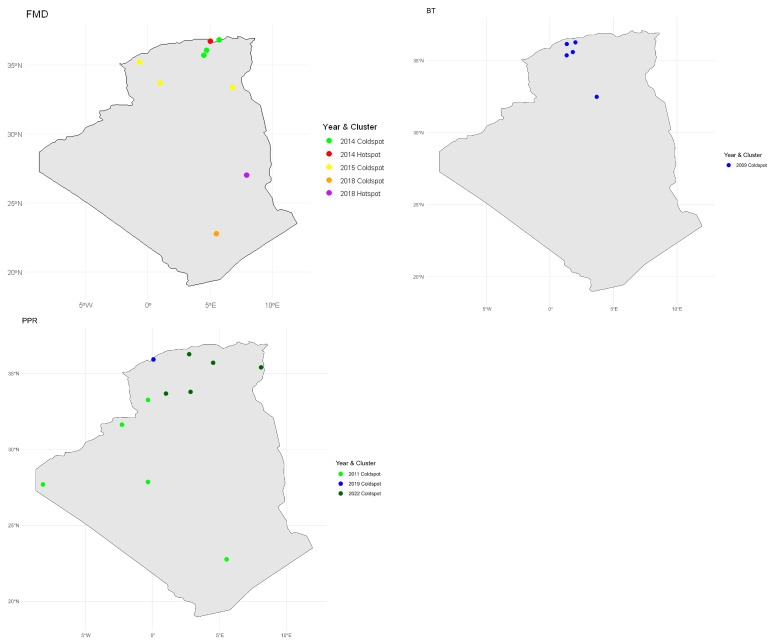
Spatial distribution of FMD, BT, and PPR incidence rates in Algeria: Identification of hotspot and coldspot clusters.

The Local Moran’s I hotspot and coldspot analysis of FMD, BT, and PPR incidences are shown in Table 2 and Figure 8.

#### 3.7.1. FMD

The Local Moran’s I analysis across multiple years revealed spatial heterogeneity in FMD incidence rates, with significant clustering identified in specific wilayas. In 2014, four regions showed significant local spatial autocorrelation: Béjaïa was identified as a hotspot (I = 0.71, *p* = 0.044), indicating a high incidence surrounded by high values. In contrast, M’Sila (I = −0.39, *p* = 0.041), Bordj Bou Arréridj (I = −0.44, *p* = 0.020), and Jijel (I = −0.56, *p* = 0.016) were coldspots, reflecting low incidence values clustered with similarly low values. In 2015, three wilayas, El Oued (I = −0.08, *p* = 0.000), El Bayadh (I = −0.92, *p* = 0.000), and Sidi Bel Abbès (I = −0.01, *p* = 0.000), also showed significant coldspot patterns. For 2018, Illizi emerged as a hotspot (I = 2.45, *p* = 0.029), while Tamanghasset was a coldspot (I = −0.19, *p* = 0.001).

No significant spatial clusters were detected in 2017, 2019, or 2022, indicating a more random or dispersed spatial distribution in those years.

#### 3.7.2. BT

The LISA analysis for the outbreak years revealed distinct local spatial patterns. In 2006, 2010, and 2011, no statistically significant local clusters were detected, indicating a random distribution of incidence rates across regions during those years. However, in 2009, five wilayas showed significant coldspots, characterized by low incidence rates surrounded by similarly low values. These included Ghardaïa (LISA I = −0.25, *p*-value = 0.00), Tiaret (LISA I = −0.10, *p*-value = 0.00), Tissemsilt (LISA I = −0.82, *p*-value = 0.00), Chlef (LISA I = −0.02, *p*-value = 0.00), and Aïn Defla (LISA I = −0.05, *p*-value = 0.00).

#### 3.7.3. PPR

The Local Moran’s I analysis revealed spatial clustering patterns of PPR incidence across different years. In 2011, five wilayas were identified as coldspots, indicating significantly low incidence rates clustered together: Tamanghasset (LISA I = −0.0019, *p* = 0), Tindouf (LISA I = −0.7228, *p* = 0), Adrar (LISA I = −0.3390, *p* = 0), Béchar (LISA I = −0.0296, *p* = 0), and Naâma (LISA I = −0.1567, *p* = 0). In contrast, 2018 showed no significant spatial clustering, with 16 wilayas classified as not significant. The year 2019 had one coldspot in Mostaganem (LISA I = −0.4314, *p* = 0.0421). Finally, in 2022, five coldspots were identified in El Bayadh (LISA I = −0.8318, *p* = 0), Laghouat (LISA I = −0.1669, *p* = 0), Tébessa (LISA I = −0.0259, *p* = 0), M’Sila (LISA I = −0.1447, *p* = 0), and Médéa (LISA I = −0.0807, *p* = 0). These results highlight the temporal and spatial variability of PPR incidence, with coldspots indicating regions of consistently low disease occurrence during outbreak years.

## 4. Discussion

This study examined the spatiotemporal dynamics of FMD, BT, and PPR in Algeria to provide insights into developing effective preventive and control measures. Our results highlight species-specific burdens, different regional hotspots for each disease, and the potential influence of climatic zones.

### 4.1. Disease Burden by Livestock Species

From 2014 to 2022, the majority of outbreaks were attributable to FMD in cattle. Conversely, sheep demonstrated a higher total number of cases despite a lower number of outbreaks. The number of outbreaks and cases observed in goats was the lowest of all species, suggesting that goats may possess either a greater resistance or a lower susceptibility/exposure to the FMD virus. This phenomenon can be attributed to the varying degrees of susceptibility exhibited by different species to FMDV. Cattle have been observed to demonstrate a higher level of vulnerability to the disease, while sheep and goats may exhibit less severe symptoms that can be undiagnosed [38]. Moreover, the presence of FMD in small ruminants is often not recognized by veterinary services in developing countries. Sheep, which frequently exhibit no symptoms, can act as reservoirs for FMD and transmit the virus to more susceptible species, such as cattle [39].

The fact that BT virus affects fewer cattle than small ruminants has already been described [40] since BT is traditionally associated with sheep, which are more vulnerable and suffer from higher mortality [41,42]. Infection in cattle and goats is usually asymptomatic or subclinical, with prolonged viremia. Furthermore, certain serotypes, such as those circulating in Algeria (1, 2, and 4), are known to be more virulent in sheep than others, such as the BTV-8, which has caused more damage in cattle on the European continent [43].

PPR has had a notable impact on sheep, which recorded the highest values of cases. However, in terms of incidence, goats were more affected. This is due to goats being more susceptible to PPRV [43]. In our study, we showed a higher incidence in sheep. The prevalence of diseases affecting goats in Algeria may be underestimated in comparison to sheep. Farmers are often more concerned about the health of sheep because of their economic importance, given that their meat is much more widely consumed and given that goats are hardier and more resistant. Furthermore, goat farming is typically practiced with very small herds of fewer than 15 animals on compact and fragile farms [44], which reduces the attention and interest of farmers and veterinarians.

### 4.2. Temporal Trend

FMD can be considered an emerging disease in Algeria [11]. Major peaks like the one in 2014 can be explained by the proactive control measures introduced after the 1999 epidemic, farm surveillance, enforcement of biosecurity protocols, and awareness campaigns targeting livestock farmers [45], as well as stamping out, compensation, and perifocal vaccination, which targeted 1.4 million cattle, 600,000 sheep, and 34,733 goats. Algeria maintained its FMD-free status for over 14 years until the 2014 outbreak, which was triggered by the illegal introduction of infected cattle from Tunisia. The same strain—O/ME-SA/Ind2001d—was identified in both countries [46,47]. Emergency control measures, including cattle vaccination and movement restrictions, were effective in containing the epidemic by 2015. However, our data show that new outbreaks later emerged in western Algeria, linked to FMD circulation in Morocco (serotype O) [48]. The absence of reported outbreaks in 2020–2021 may reflect the effectiveness of the post-2018 control measures or, alternatively, an under-reporting bias due to limitations in passive surveillance. This is consistent with the resurgence of approximately 40 outbreaks reported in 2022 across 11 wilayas.

Our findings indicate that FMD remains endemic in Algeria, with no consistent temporal pattern or clearly identified drivers. Several factors may contribute to its persistence and re-emergence: (1) insufficient vaccination coverage—particularly in remote or underserved areas—leaving susceptible animal populations [49,50]; (2) persistence in the environment [51]; (3) potential wildlife reservoirs—a hypothesis that requires further investigation in Algeria [52]; (4) uncontrolled movement of infected animals; (5) viral evolution—high mutation rates may lead to the emergence of novel strains not covered by current vaccines [53]; (6) climatic conditions, as temperature and humidity have been identified as key factors in viral survival and transmission [54]; (7) human-related practices—such as improper carcass disposal, poor biosecurity, and unsafe handling of infected animals; and (8) the presence of asymptomatic carriers—infected or vaccinated ruminants may enter a persistent phase, shedding low levels of FMDV in oropharyngeal fluid for months to years [55,56]. This carrier state is especially relevant in Algeria, where total culling is rarely practiced, allowing infected animals to survive and potentially spread the virus. Recently, new FMD outbreaks have been reported, including the emergence of serotype SAT2 in 2024, highlighting the continued threat to animal health and the urgent need to reassess and strengthen current prophylactic strategies.

Temporal trends of BT indicated that the first outbreak (BTV-2) was reported in July 2000, originating in Tunisia and initially affecting the northeast before spreading throughout the country [57]. In 2006, BTV-1 (of sub-Saharan African origin) was recorded in sheep and continued to spread in the steppe region, and between 2009 and 2011, outbreaks were reported more frequently in sheep than in cattle. These were caused by the re-emergence of BTV-1 (2009) and BTV-4 (2010), with unknown origins [26]. BT has resurfaced after a prolonged absence, largely attributable to the year-round presence of adult *Culicoides imicola* in the region [58]. This phenomenon is sustained by the persistence of suitable eco-climatic niches, particularly in the context of ongoing climate change [59]. Another explanation may be the presence of multiple serotypes within the same geographical area, as it has been noted that BTV demonstrates prolonged viability in regions where multiple serotypes of the virus are present [42].

The decline and eventual disappearance of BT outbreaks in Algeria can be largely attributed to targeted surveillance, isolation of infected animals, and vector control measures. However, vaccination campaigns have not been widely implemented. This is partly due to the limited cross-protective efficacy of conventional vaccines across different BTV serotypes [60], as well as the varying virulence and clinical expression of BT among ruminant species [40]. For instance, Greece achieved BT-free status in the 1990s through zoo-sanitary measures alone, without vaccination. Conversely, Italy adopted an active vaccination strategy using attenuated vaccines but continues to report sporadic outbreaks, partly due to the circulation of both field and vaccine-derived virus strains [61]. Tunisia ceased its vaccine-based BT control strategy in 2017 [62,63]. Globally, vaccination strategies differ depending on the target species, national regulations, and vaccine characteristics, each with specific advantages and limitations. Recent advances have led to the development of next-generation vaccines, including subunit vaccines based on VP2 or virus-like particles, viral vectors expressing BTV proteins, and reverse-genetics-based platforms, which promise broader protection with fewer side effects [64,65]. Algeria’s decision not to vaccinate livestock against BT likely reflects cost–benefit considerations related to these new technologies. The absence of reported cases since 2020 has temporarily validated this approach.

PPR is endemic in Algeria, with nine outbreaks reported over the past 15 years in our database. Initial outbreaks occurred in the south between 2010 and 2011, followed by cases in central Algeria (Ghardaïa) during 2012–2013, likely linked to the introduction of lineage IV of the virus from Morocco, where outbreaks were reported in 2008 [10]. In response, Algerian veterinary authorities administered 200,000 doses of the Nig. 75/1 vaccine strain [66]. The major peak observed in 2018–2019 can be attributed in part to unfavorable climatic conditions. In 2019, Algerian weather stations recorded rainfall deficits ranging from −5% to −35% in the west, −10% to −31% in the south (e.g., M’Sila, Bousaada), and about −7% in the southeast (e.g., Biskra). These drought conditions likely prompted increased transhumance in search of water and pasture, facilitating the spread of the virus among naïve animal populations [67]. Mixed herds of sheep and goats, which are in close and constant contact, are particularly susceptible to severe outbreaks [68]. This leads to epizootic waves that can subsequently become endemic once the virus circulates through a region, a pattern observed in many African countries [69].

Additionally, vaccination coverage has remained insufficient. Only 200,000 doses were administered in 2011, despite a national small ruminant population exceeding 29 million sheep and nearly 5 million goats. This has left a significant proportion of animals unprotected and vulnerable to new incursions of the disease.

### 4.3. Spatial Distribution

Six northeastern wilayas were identified as highly endemic for FMD, and they also rank among Algeria’s most important livestock regions. For example, Sétif has the highest cattle density in the east, while Bouira and Tizi Ouzou host major slaughterhouses and livestock markets. Béjaia, identified as a hotspot in 2014, likely played a pivotal role in the spread of FMD due to its proximity to Sétif—where the outbreak began—and its large livestock market. Farmers, fearing impending movement restrictions, hastily sold or exchanged potentially infected animals, accelerating disease dissemination.

In 2018, Illizi emerged as a new FMD hotspot, particularly for small ruminants (2481 cases in 2018; 3369 in 2019). Although El Oued reported the first cases, Illizi functions as a major transit zone for small ruminants. Its proximity to military bases (e.g., Hassi Messaoud) creates demand for meat, and its location near the eastern border facilitates informal trans-Sahelian trade in livestock, including camels. This cross-border movement has contributed to the spread of epizootics, such as the 1999 serotype O outbreak, linked to animals from Mali and Mauritania [70]. Algeria’s high “eigenvector” centrality highlights its vulnerability to transboundary epizootic incursions from neighbouring countries [71]. The absence of statistically significant spatial clustering in FMD cases likely results from two main factors: the virus’s extreme contagiousness and the constant movement of animals across regions.

In contrast, BT displayed spatial heterogeneity, with outbreaks mainly affecting the steppe and eastern border regions. These areas combine high livestock densities with favorable environmental conditions for vector survival (e.g., temperature and humidity) [72].

PPR cases were more frequent in northern and north-Saharan zones (e.g., El Oued, Ghardaïa, Biskra), but no true hotspots were identified, suggesting widespread virus circulation due to continuous animal movement. This pattern reflects the role of transhumance and mixed-species herding (sheep and goats) in facilitating disease transmission [14,67]. The vast Saharan wilayas of Tamanrasset and Adrar showed lower PPR activity (“coldspots”), likely due to extensive grazing practices that reduce animal density and contact. The coldspots observed in 2022 may also indicate the effectiveness of past vaccination campaigns. In 2020 and 2021, over 6 and 7 million animals were vaccinated, respectively, with priority given to high-density regions such as El Bayadh, Laghouat, M’sila, and Tébessa.

However, several structural challenges hinder control efforts. Algeria shares 6343 km of land borders with seven countries, and the lack of strict biosecurity and border control facilitates cross-border transmission. Weekly livestock markets (souks), operating without proper veterinary oversight, add to the risk. Inadequate or irregular vaccination programs, insufficient vaccine supplies, and improper administration further weaken control measures. For PPR, the thermolability of the vaccine [73] necessitates a robust cold chain, which remains a challenge. Moreover, the absence of a compensation scheme for PPR discourages farmers from reporting cases—unlike FMD, brucellosis, or tuberculosis, for which farmers receive up to 80% compensation. This likely leads to under-reporting and underestimation of true disease incidence [74]. Outbreaks reported in Algiers (October 2015) and northwestern Morocco (June 2015) [75] underscore the risk of PPRV spreading to Europe, especially through uncontrolled animal movements [14].

### 4.4. Bioclimatic Associations with Disease Outbreaks

Our spatial analysis identified the sub-humid and semi-arid zones as hotspots for FMD and BT across all species. This distribution is likely associated with both a high concentration of ruminant farms and favorable climatic conditions. Humidity in sub-humid areas may promote FMDV transmission [11], while the ecology of BT vectors is constrained by the high temperatures and aridity of the Saharan zones [72]. For PPR, the majority of cases and outbreaks occurred in the semi-arid and Saharan zones, which host the country’s most important goat-breeding areas. These regions are also affected by cross-border movements of animals, including from neighboring countries where PPR is endemic. Notably, outbreaks in goats were also concentrated in humid and sub-humid zones, suggesting that intensive farming systems—characterized by confinement and close animal contact—may facilitate PPRV transmission. In general, disease notifications were more frequent in densely populated areas, such as the humid and sub-humid wilayas.

However, these descriptive findings must be interpreted cautiously. Given that data originate from passive surveillance systems, which are typically affected by under-reporting [76], observed patterns may reflect higher detection rather than true disease burden. A more accurate understanding of disease dynamics in Algeria will require future studies to estimate under-reporting levels by region and proximity to reporting centers, allowing for correction of the observed data patterns.

## 5. Recommendations for Prevention and Control of FMD, BT, and PPR in Algeria

Despite periodic control measures, our findings suggest that FMD, BT, and PPR remain endemic in Algeria. This reinforces the country’s central role in regional epizootics and highlights major gaps in disease prevention. Weak coordination, under-reporting, and insufficient veterinary infrastructure—especially in the south—are major constraints. High livestock density and uncontrolled animal movement, driven by both legal and illegal trade and grazing practices, further facilitate disease spread. Based on our findings, we recommend:

- Species-specific local surveillance and control: Target interventions in areas with the highest disease burden. Current vaccination strategies should be re-evaluated in terms of cost-effectiveness and geographic targeting.
- Cross-border collaboration: As TADs readily spread across national borders, joint surveillance and harmonized vaccination campaigns with neighboring countries are essential.
- Climatic and environmental data integration: Incorporate these data into predictive models, especially for vector-borne diseases like BT, to improve forecasting and preparedness.
- Molecular epidemiology and wildlife studies: Further investigation is needed into viral evolution and the role of wildlife reservoirs in disease transmission.
- Advanced spatiotemporal modeling: Use predictive models to identify high-risk areas and timeframes, enabling efficient, timely, and evidence-based interventions.

## Figures and Tables

**Table 1:** Results of the Negative Binomial Regression Model assessing factors associated with FMD, PPR, and BT incidence in Algeria.

	Variable	SE	IRR	IRR 95% CI	*p* Value
FMD	Year (2014)	19.55	29.08	6.75–96.70	<0.0001
	Year (2018)	13.78	17.47	3.51–78.45	<0.001
	Species (Cattle)	4.42	12.57	4.19–36.69	<0.0001
	Species (Sheep)	5.31	15.67	7.76–31.34	<0.001
	Quarter (2)	27.64	16.83	0.69–1492.24	0.086
PPR	Quarter (4)	1.79	3.25	0.98–9.04	0.033
	Quarter (1)	1.97	3.81	1.21–9.67	0.010
	Species (Sheep)	1.31	4.32	2.35–7.88	<0.001
BT	Quarter (3)	0.03	0.03	0.00–0.34	0.006
	Quarter (4)	0.02	0.02	0.00–0.23	0.0021
	Species (Sheep)	162.68	155.53	20.02–1208.25	<0.0001

SE, Standard Error; IRR, Incidence Rate Ratio; IRR 95% CI, 95% of IRR Confidence Interval.

**Table 2:** Local Moran’s I hotspot and coldspot analysis of FMD, BT, and PPR incidences.

Disease	Year	Wilaya	Latitude	Longitude	Incidence Rate	LISA I	*p*-Value	Cluster
FMD	2018	Tamanghasset	22.7697	5.548	0.49	−0.19	0.001	Coldspot
	2018	Illizi	26.9997	7.9707	6.91	2.45	0.029	Hotspot
	2015	El_Oued	33.3714	6.8554	0.01	−0.08	0.000	Coldspot
	2015	El_Bayadh	33.6829	1.0217	0.06	−0.92	0.000	Coldspot
	2015	Sidi_Bel_Abbès	35.2007	−0.6323	0.02	−0.01	0.000	Coldspot
	2014	M’Sila	35.7142	4.5422	0.24	−0.39	0.041	Coldspot
	2014	Bordj_Bou_Arréridj	36.0774	4.7611	0.25	−0.44	0.020	Coldspot
	2014	Béjaïa	36.7222	5.0503	2.03	0.71	0.044	Hotspot
	2014	Jijel	36.8321	5.7617	0.12	−0.56	0.016	Coldspot
PPR	2011	Tamanghasset	22.7697	5.548	0.13	0.00	0.000	Coldspot
	2011	Tindouf	27.7	−8.1528	0.32	−0.72	0.000	Coldspot
	2011	Adrar	27.8668	−0.3196	0.02	−0.34	0.000	Coldspot
	2011	Béchar	31.6348	−2.2641	0.18	−0.03	0.000	Coldspot
	2011	Naâma	33.2672	−0.3196	0.06	−0.16	0.000	Coldspot
	2022	El_Bayadh	33.6829	1.0217	0.04	−0.83	0.000	Coldspot
	2022	Laghouat	33.797	2.8558	0.00	−0.17	0.000	Coldspot
	2022	Tébessa	35.4088	8.1155	0.02	−0.03	0.000	Coldspot
	2022	M’Sila	35.7142	4.5422	0.00	−0.14	0.000	Coldspot
	2019	Mostaganem	35.927	0.0872	0.11	−0.43	0.042	Coldspot
	2022	Médéa	36.2641	2.7539	0.00	−0.08	0.000	Coldspot
BT	2009	Ghardaïa	32.4921	3.6776	0.01	−0.25	0.00	Coldspot
	2009	Tiaret	35.371	1.3169	0.02	−0.10	0.00	Coldspot
	2009	Tissemsilt	35.6015	1.799	0.12	−0.82	0.00	Coldspot
	2009	Chlef	36.1596	1.3259	0.06	−0.02	0.00	Coldspot
	2009	Aïn_Defla	36.2741	2.0017	0.03	−0.05	0.00	Coldspot

## Data Availability

All relevant data are within the manuscript and its Supporting Information Files.

## Supplementary Materials

The following supporting information can be downloaded at: https://www.mdpi.com/article/10.3390/v17071008/s1, Table S1. Raw data; Table S2. Annual incidences of FMD, BT, and PPR per 1000 animals; Figure S1. Spatiotemporal distribution of FMD outbreaks in cattle across Algeria between 2014 and 2022; Figure S2. Spatiotemporal distribution of FMD outbreaks in sheep across Algeria between 2014 and 2022; Figure S3. Spatiotemporal distribution of FMD outbreaks in goats across Algeria between 2014 and 2022; Figure S4. Spatiotemporal distribution of BT outbreaks in sheep across Algeria between 2006 and 2020; Figure S5. Spatiotemporal distribution of BT outbreaks in cattle across Algeria between 2006 and 2020; Figure S6. Spatiotemporal distribution of BT outbreaks in goats across Algeria between 2006 and 2020; Figure S7. Spatiotemporal distribution of PPR outbreaks in sheep across Algeria between 2011 and 2022; Figure S8. Spatiotemporal distribution of PPR outbreaks in goats across Algeria between 2011 and 2022.
