# Supplementary material for: Spatiotemporal Dynamics of Emerging Foot-and-Mouth Disease, Bluetongue, and Peste Des Petits Ruminants in Algeria
Source: Viruses. 2025 Jul 17;17(7):1008. doi: 10.3390/v17071008 (PMC12300541; doi:10.3390/v17071008)
Supplement: Supplementary file 1 [file viruses-17-01008-s001.zip › viruses-3637953-supplementary figures.pdf]

Article

Spatiotemporal Dynamics of Emerging Foot-and-Mouth Disease, Bluetongue, and Peste Des Petits Ruminants in Algeria

Ilhem Zouyed [1], Sabrina Boussena [2], Nacira Ramdani [3], Houssem Eddine Damerdji [1], Julio A. Benavides [4,5] and Hacène Medkour [4,*]

[1] Institute of Veterinary Sciences, University Constantine 1 Frères Mentouri, Constantine 25100, Algeria; ilhem.zouyed@umc.edu.dz (I.Z.); houss.dm@gmail.com (H.E.D.)
[2] Management of Animal Health and Productions Laboratory, University Constantine 1 Frères Mentouri, Constantine 25100, Algeria; s_boussena@umc.edu.dz
[3] Regional Veterinary Laboratory of El Oued, National Institute of Veterinary Medicine, El Oued 39000, Algeria; drnaciraramdani@gmail.com
[4] MIVEGEC, University of Montpellier, IRD, CNRS, Montpellier 34394, France; julio.benavides@ird.fr (J.A.B.); hacene.medkour@ird.fr (H.M.)
[5] Doctorado en Medicina de la Conservación y One Health Institute, Facultad de Ciencias de la Vida, Universidad Andrés Bello, Santiago 8320000, Chile; julio.benavides@ird.fr
* Correspondence: hacene.medkour@ird.fr

Supplementary figures

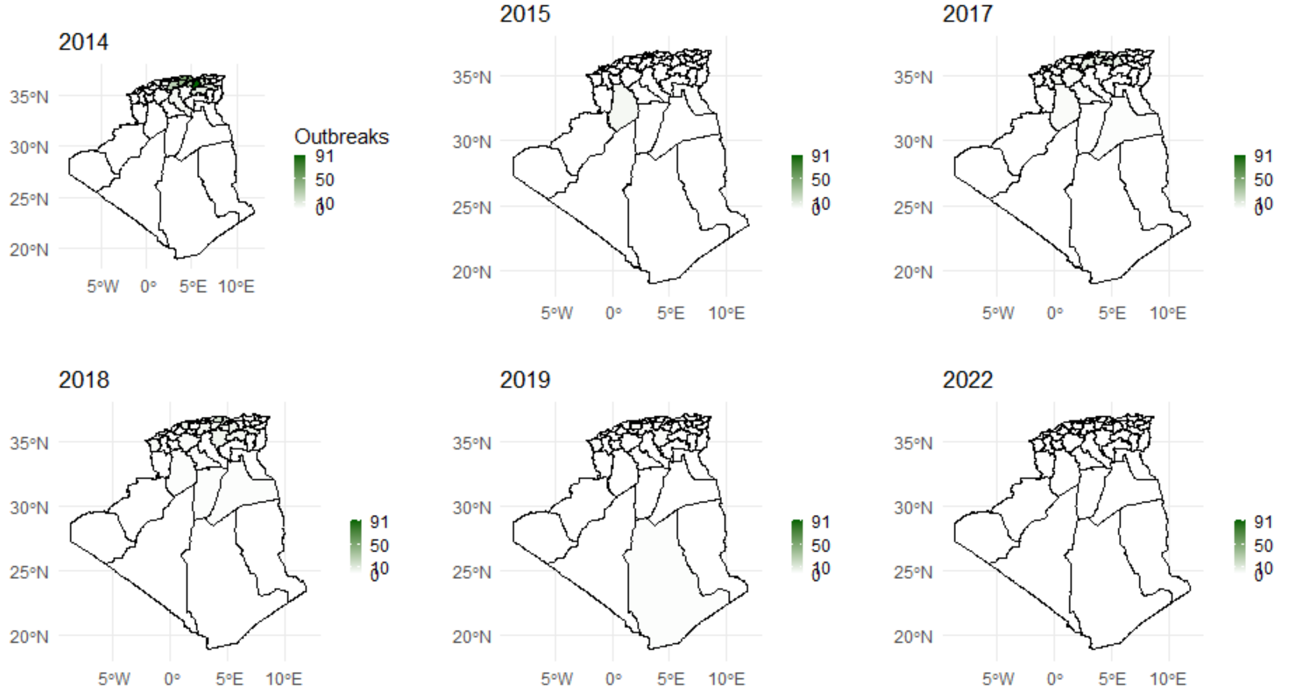

Figure S1. Spatio-temporal distribution of FMD outbreaks in cattle across Algeria between 2014 and 2022.

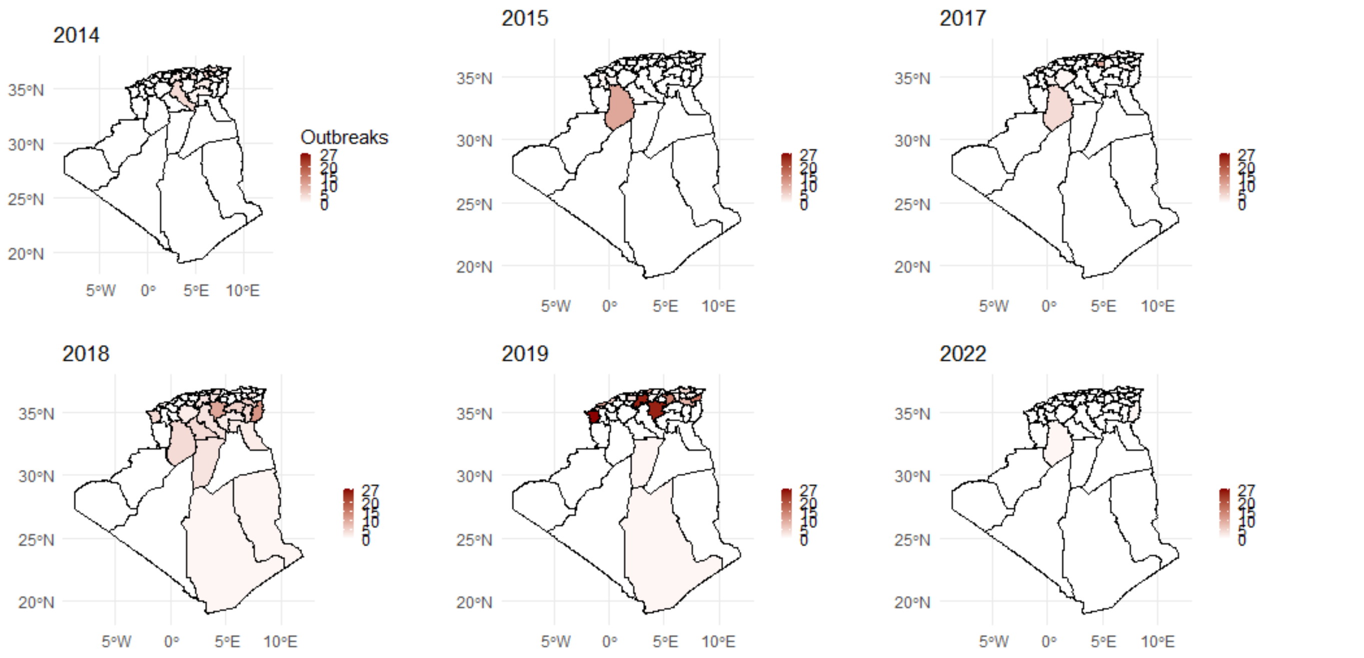

Figure S2. Spatio-temporal distribution of FMD outbreaks in sheep across Algeria between 2014 and 2022.

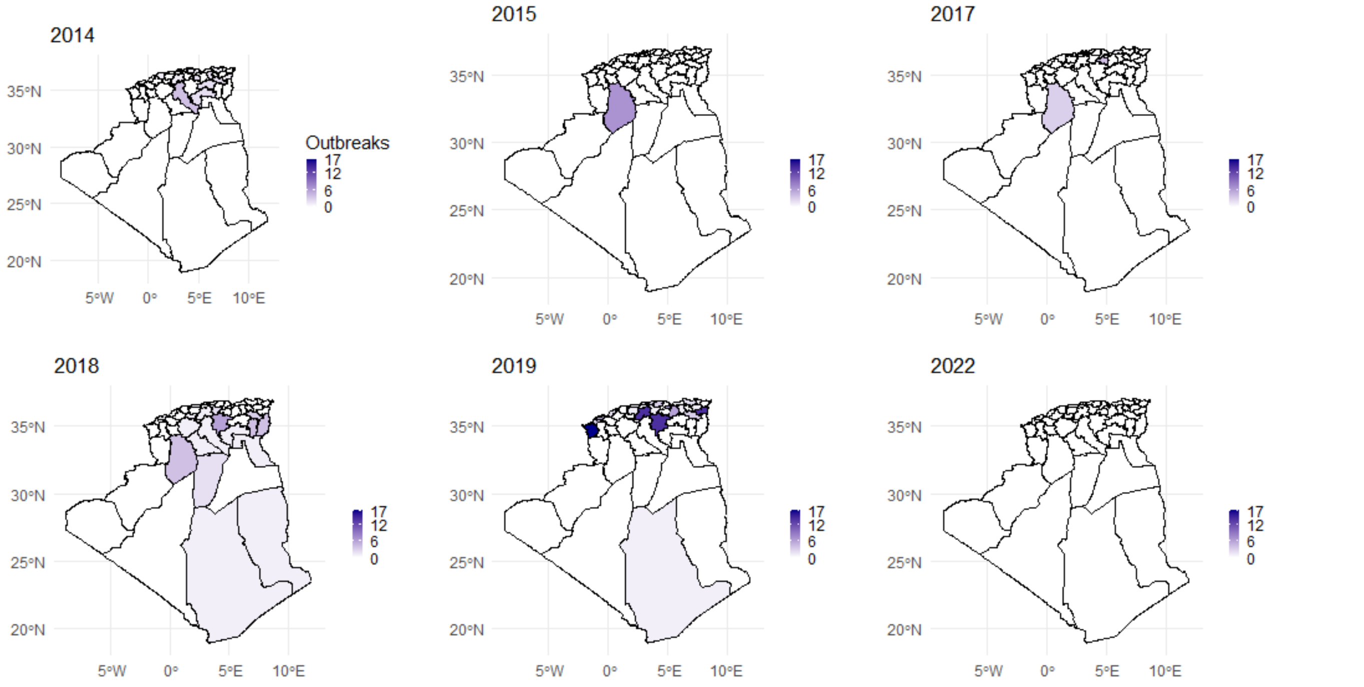

Figure S3. Spatio-temporal distribution of FMD outbreaks in goats across Algeria between 2014 and 2022.

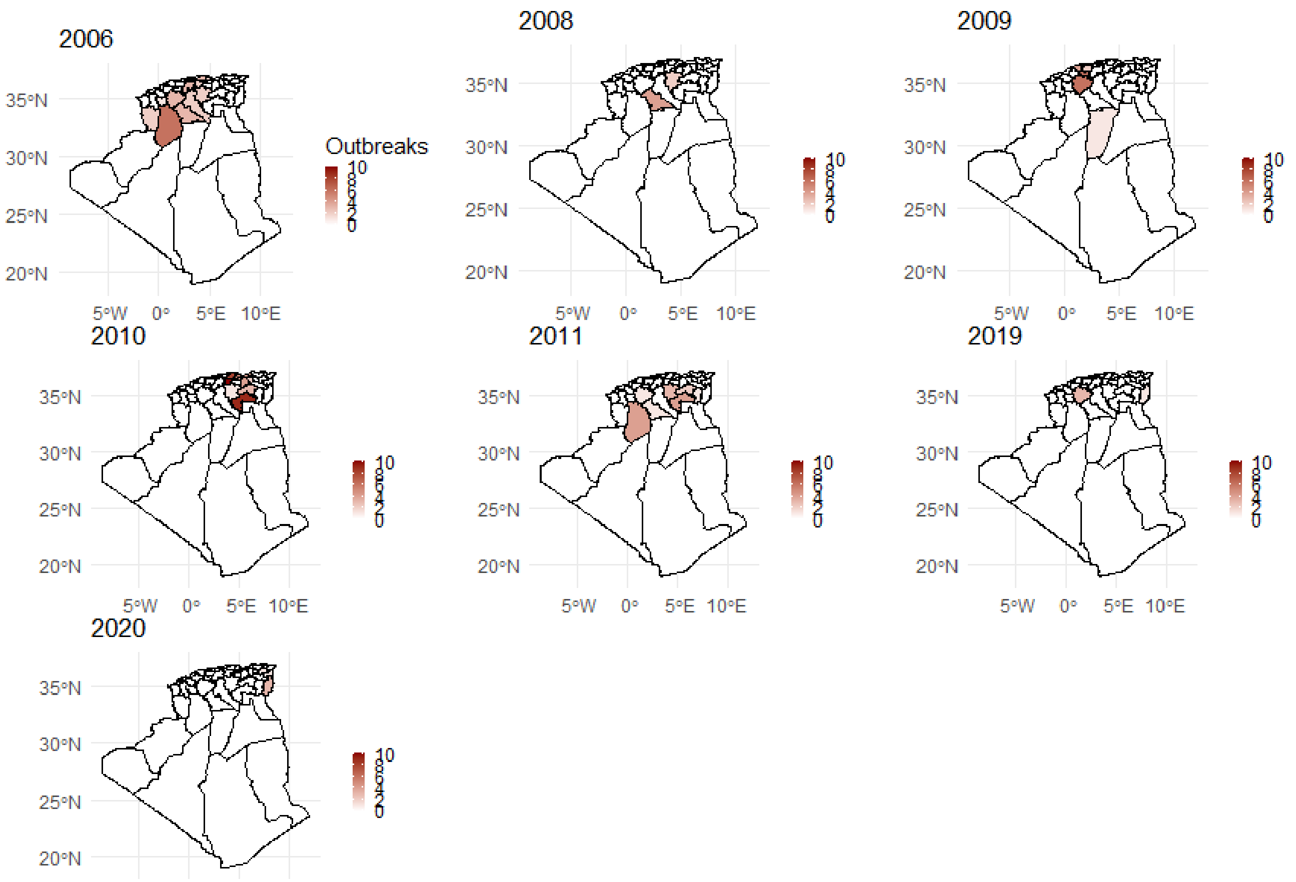

Figure S4. Spatio-temporal distribution of BT outbreaks in sheep across Algeria between 2006 and 2020.

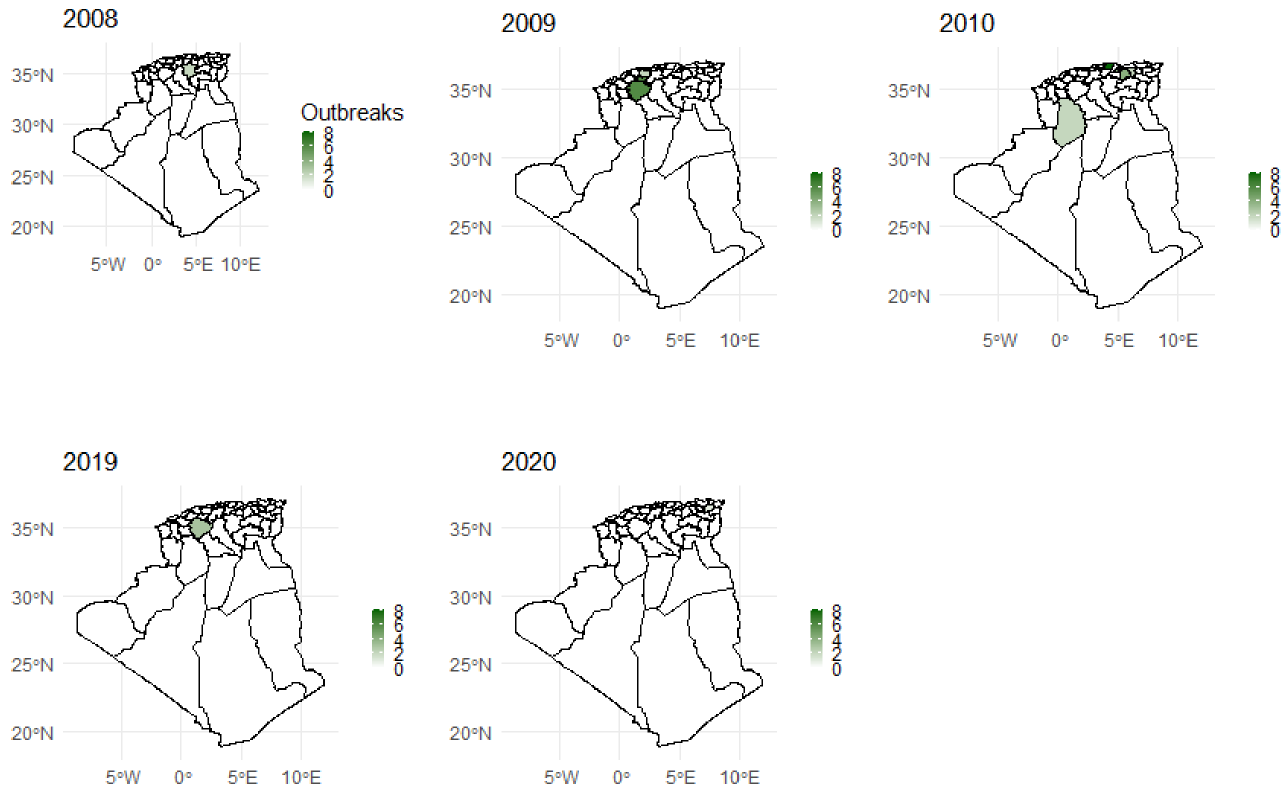

Figure S5. Spatio-temporal distribution of BT outbreaks in cattle across Algeria between 2006 and 2020.

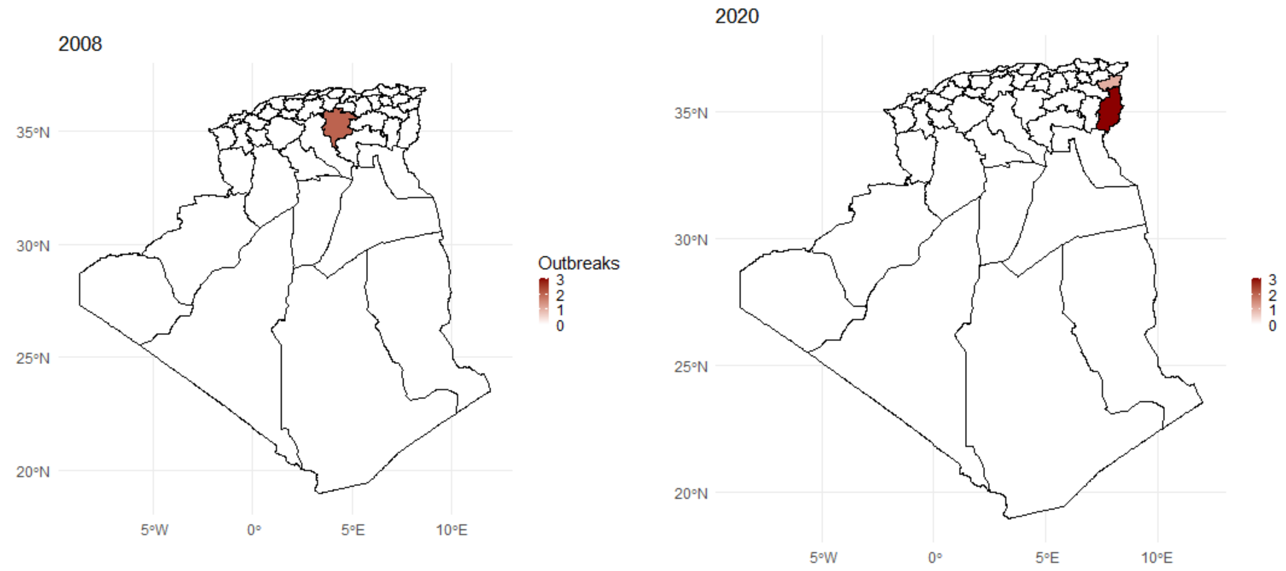

Figure S6. Spatio-temporal distribution of BT outbreaks in goats across Algeria between 2006 and 2020.

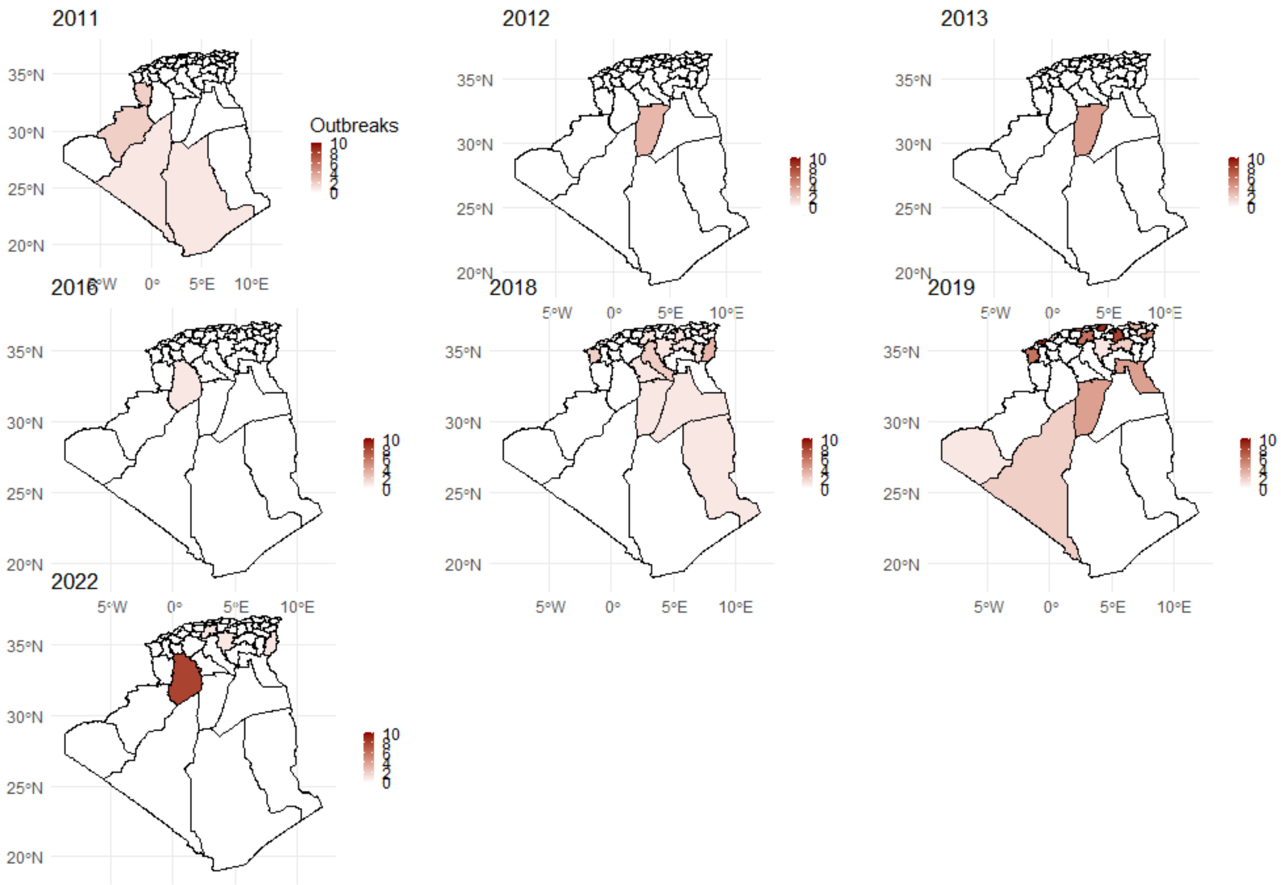

Figure S7. Spatio-temporal distribution of PPR outbreaks in sheep across Algeria between 2011 and 2022.

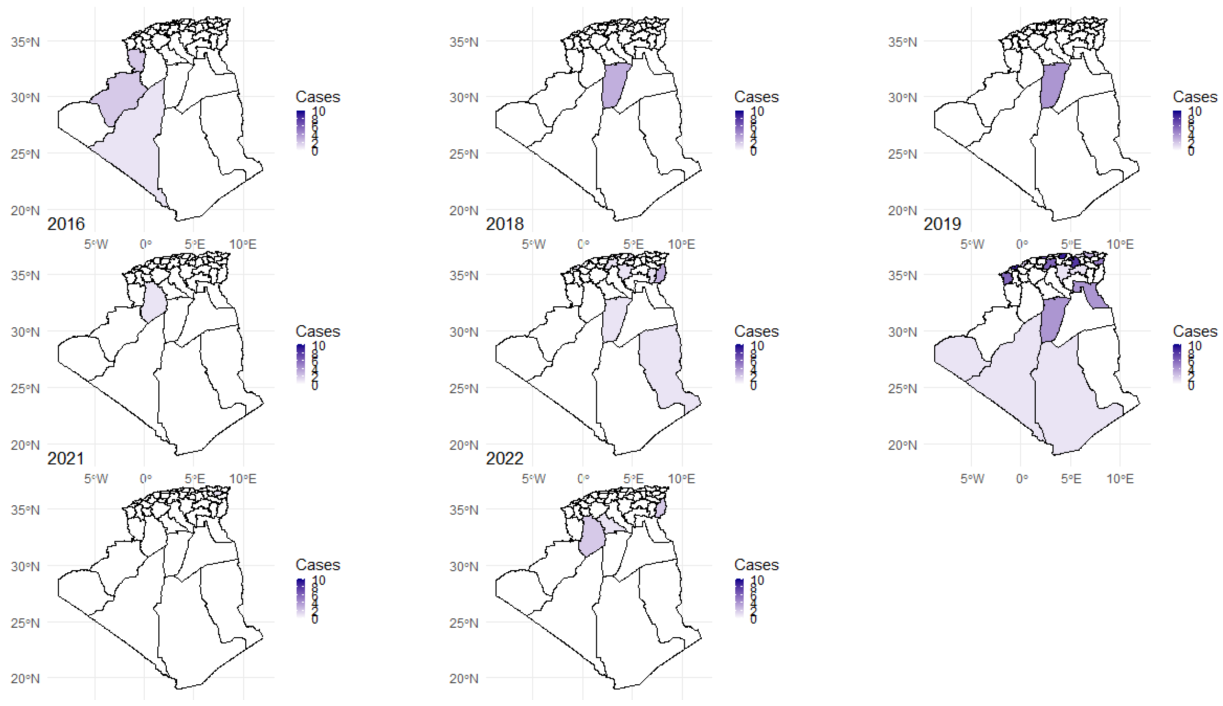

Figure S8. Spatio-temporal distribution of FMD outbreaks in goats across Algeria between 2006 and 2020.
